# Clozapine-induced parotitis: A case study

**DOI:** 10.1192/j.eurpsy.2021.1433

**Published:** 2021-08-13

**Authors:** M. Kacem, H. Mami, S. Sellami, M. Moalla, S. Ben Frej, R. Bouzid

**Affiliations:** 1 Department Of Psychiatry, University hospital of mahdia, chebba, Tunisia; 2 Department Of Psychiatry, University hospital of Nabeul, Nabeul, Tunisia; 3 Department Of Psychiatry, University Hospital Of Mahdia, Tunisia., Psychiatry, Mahdia, Tunisia

**Keywords:** clozapine, induced, parotitis, swelling

## Abstract

**Introduction:**

Clozapine is the drug of choice for patients with an unsatisfactory response to classic antipsychotic treatment. Little is known about the involvement of clozapine in the development of parotid disease.

**Objectives:**

Identify the clinical characteristics of Clozapine-induced parotitis through a case and literature review.

**Methods:**

We report the case of a patient with a refractory schizoaffective disorder, bipolar type and who developed recurrent parotitis while taking clozapine. We conducted a literature review based on a PubMed search of articles published on this subject with the following keywords: ‘parotitis clozapine’.

**Results:**

Miss W., 34 years old, suffers from a severe schizoaffective disorder that has been diagnosed for several years. She has received various psychotropic medications. She suffered from frequent relapses that required recurrent hospital admissions. One year ago, a diagnosis of treatment-resistant schizoaffective disorder was made. The decision to introduce clozapine, associated with mood stabilizer treatment, was made on the basis of her treatment refractory symptoms. She experienced considerable sialorrhea after beginning clozapine treatment. Miss W. developed bilateral recurrent swelling over both temporal-mandibular areas after 6 months of treatment. It often appears after eating and lasts from 4 to 6 hours. There was no change in white blood cell count and she was afebrile. An otolaryngologist was consulted and a diagnosis of clozapine-induced parotitis was suggested. A spasmolytic and an anticholinergic treatment were prescribed and clozapine was continued.

**Conclusions:**

This iatrogenic effect of clozapine must be recognized by clinicians in order to be better prevented.

